# Cardio-oncology: a new medical issue

**DOI:** 10.3332/ecancer.2008.126

**Published:** 2008-12-15

**Authors:** D Cardinale, A Colombo, G Lamantia, N Colombo, M Civelli, G De Giacomi, C Pandini, MT Sandri, CM Cipolla

**Affiliations:** Cardiology Unit and Laboratory Medicine Unit, European Institute of Oncology, IRCCS, 20141 Milan, Italy

## Abstract

Due to the increasing number of long-term cancer survivors, the ageing of the population, as well as the increased incidence and prevalence of oncologic and cardiovascular diseases, the number of patients presenting oncologic and cardiologic co-morbidities are increasing. Accordingly, there is a rapidly growing need for a comprehensive and proficient management of patients in whom the two co-morbidities exist, and for cancer patients whose clinical history and oncologic treatment put them at higher risk for developing cardiovascular problems, in order to provide the optimal treatment in every situation, and to avoid the possibility that the development of the second disease does not lead to a reduction of therapeutic opportunities for the patient. A new discipline, c***ardio-oncology***, has been created to deal with this need. Its aim is to investigate new strategies, collect new evidence-based indications and develop interdisciplinary expertise in order to manage this growing category of patients. Cardio-oncology deals with the following main clinical and research areas: early diagnosis of cardiotoxicity, risk stratification and preventions, treatment and monitoring of cardiotoxicity.

The survival rate of patients with cancer, as well as those with cardiovascular disease has greatly increased over the past three decades [1,2,3]. This is partly due to improvements in pharmacological treatment and in surgery procedures and because of the reduction and control of major risk factors. On the other hand, due to the ageing of the population, the incidence and prevalence of oncologic and cardiovascular disease, as well as the number of patients presenting oncologic and cardiologic co-morbidities, are increasing.

Because of overlapping risk factors, such as obesity, hormone replacement therapy and, in particular, smoking, heart disease patients are likely to have a higher risk of cancer than the general population [4,5].

Conversely, the development of effective prevention screening and treatment strategies for many cancers, particularly in the early stages of the disease, has resulted in an enormous population of long-term cancer survivors. According to estimates from the National Cancer Institute and the Centres for Disease Control and Prevention, there were more than ten million cancer survivors in the United States alone in 2002 [6]. Many of these survivors have had radiation or chemotherapy (CT) treatments, with potential long-term cardiovascular toxicities, that may ultimately attenuate the clinical success of oncologic treatments. Data from recent oncology literature indicate that more than half of all patients exposed to chemotherapy will show some degree of cardiac dysfunction ten to 20 years after CT, 5% will develop overt heart failure, and 40% will experience arrhythmias [7]. This population shows an eightfold higher cardiovascular mortality when compared to the general population [8].

For these reasons, there is a rapidly growing need for comprehensive and professional management aimed at patients in whom the two co-morbidities exist, and at cancer patients whose clinical history and oncologic treatment put them at higher risk for developing cardiovascular problems. This must be accomplished in order to provide optimal treatment in every situation, and to avoid the possibility that the onset of a second disease may lead to a reduction of therapeutic opportunities and negative long-term results.

Indeed, when a cardiac patient develops an oncological problem, the cardiologist often loses interest in him or her and tends to inherit a defeatist attitude, which may exclude the patient from other intensive treatment and/or intervention possibilities. Conversely, when a cancer patient develops a cardiologic problem, he/she is invariably excluded from first-line, more aggressive (and therefore, more effective) therapeutic strategies, negatively impacting his oncologic outcome.

The final result is that this patient goes beyond the jurisdiction of both the cardiologist and the oncologist, and there is no one who takes it upon himself to give this patient comprehensive care. As a consequence, the management of such patients is limited, disjointed and often inadequate. The patient feels left alone and unprotected. This behaviour may lead to negative prognostic influence during the course of the two illnesses, whereas, under different circumstances, the patient may have been effectively treated.

In order to deal with this need, a new discipline, ***cardio-oncology***, has been created. Its aim is to investigate innovative strategies, collect evidence-based indications, and to develop interdisciplinary expertise, which will be able to manage this new and growing category of patients, to guarantee correct clinical administration, and to provide the best therapeutic opportunities, also in terms of the impact on prognosis of the two concomitant diseases, for these more complex patients.

## Diagnosis of cardiotoxicity

Cardiotoxicity is a common complication of CT. The clinical manifestation of CT can range, in its more typical form, chronic cardiotoxicity, from transient asymptomatic left ventricular dysfunction to cardiac death [9,10].

This is a growing problem in the setting of clinical oncology, given the increasing number of long-term cancer survivors, the tendency to use progressively higher doses of anthracyclines (AC), the introduction of new anti-tumour agents with possible cardiotoxic properties and combined treatments with synergistic harmful effects [10–13]. The clinical implications of cardiotoxicity are particularly relevant in those cancer patients in whom the onset of cardiac dysfunction, even asymptomatic, seriously limits their therapeutic opportunities and negatively impacts their clinical outcome. Therefore, early identification of patients at risk for cardiotoxicity represents a primary goal for both cardiologists and oncologists, allowing for the definition of personalized antineoplastic therapeutic strategies and/or interventions [14].

To detect CT-induced cardiac damage in an early phase, regular cardiac function assessment is, at present, recommended by oncologic guidelines. Cardiologic surveillance is required during CT, to allow for the administration of the highest dose without inducing cardiac injury, and after completion of the CT to identify cardiac damage at an early preclinical stage. This is carried out in order to limit, by means of pharmacologic intervention, the progression of cardiac dysfunction.

At least three international consensus guidelines recommend evaluation of left ventricular ejection fraction (LVEF) at the beginning of antineoplastic therapy, after administration of half the total anthracyclines cumulative dose, and before every following dose [15–17]. It is also recommended during follow-up, and LVEF evaluation at three, six, and 12 months after the end of treatment [10,18]. A decline of LVEF by more than 10%, associated to an absolute LVEF value <50%, is suggested as a criterion for suspending treatment [19].

The weak point of such an approach is that it is too late. In fact, cardiac damage is usually detected when functional impairment has already occurred [20,21]. Many doubts have been raised about the utility of monitoring the cardiac function by LVEF evaluation by means of most utilized methods in clinical practice, including echocardiography and radionuclide-angiocardiography, since the value of this monitoring seems to be neither sensitive nor specific enough to give an early prediction of the development of cardiac dysfunction after CT. Actually, it permits the identification of cardiac damage only after the onset of cardiac dysfunction, not allowing for any early strategy able to prevent future cardiomyopathy (CMP) [20]. On the other hand, the evidence of unaffected heart function does not exclude the possibility of further cardiac deterioration [22–24].

Hence, there is growing expectation for newer, non-invasive and cost-effective diagnostic tools for the early identification of patients prone to developing drug-induced cardiotoxicity.

In our clinical practice, we utilize different markers for the early identification of patients at increased risk of cardiotoxicity: troponin I and N-terminal-proB-type natriuretic peptide (NT-proBNP). For both of these, an accurate predictive value has been demonstrated by our recent investigations.

## Markers of myocardial damage: troponin I

Troponin I (TnI) is a protein present exclusively in the myocardial cells. The TnI plasma concentration is a well-established, specific and sensitive marker of myocardial injury, widely used for the diagnosis and the risk stratification of acute coronary syndromes. More recently, TnI has been utilized to detect cardiac damage in other clinical settings, such as heart failure, acute pulmonary embolism, renal failure, sepsis and septic shock and stroke [25,26]. Evidence of troponin’s release after CT has previously been demonstrated in animal models and in clinical studies [27,28].

**Figure 1:**
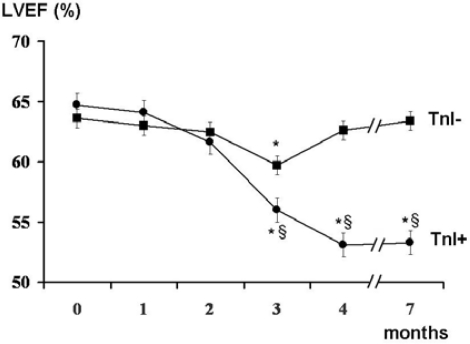
Left ventricular ejection fraction (LVEF) at baseline and during the seven months of follow-up of troponin I positive (TnI+) and negative (TnI-) patients

**Figure 2:**
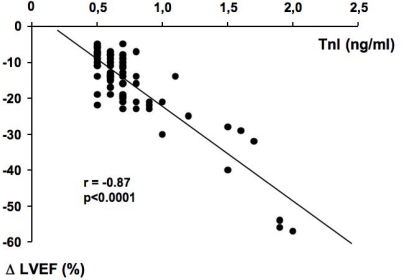
**Scatterplot of left ventricular ejection fraction (LVEF) changes against troponin I (TnI) value in TnI+ patients. Modified from Cardinale *et al* [29]** * = p<0.001 versus baseline (month 0); fl = p<0.001 versus TnI-group. Data are shown as mean ±95% CI.

In previous studies, we demonstrated that TnI is a sensitive and specific marker of myocardial injury after high-dose CT, and that it is able to predict, in a very early phase, the development of future ventricular dysfunction, as well as its severity [29,30]. Indeed, patients showing an increase of this marker soon after the end of CT significantly decreased systolic ventricular function in the months following (Figure 1). Moreover, in our studies, we found a strong relationship between the maximal TnI value measured soon after CT and the degree of LVEF reduction during the follow-up (Figure 2).

**Figure 3:**
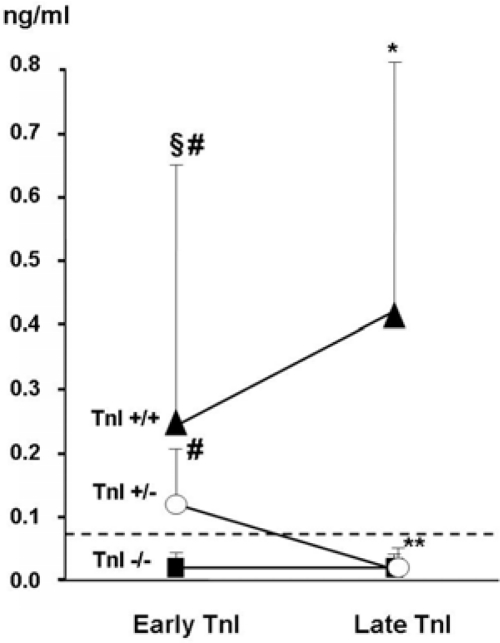
**Early and late troponin I (TnI) values in the three study groups** *p<0.05 versus early TnI; **p<0.001 versus early TnI; fl=p<0.001 versus TnI+/−; #p<0.001 versus TnI−/−. From Cardinale *et al* [31]

In a more recent work, a large population with a long follow-up (48 months) and a wide spectrum of cardiac events was considered [31]. As in the previous studies, we evaluated TnI at baseline and in the first 72 hours after CT (early TnI); in addition, in all patients, we also measured TnI one month later (late TnI). Three different TnI patterns were identified in our population (Figure 3): in most patients (70%), an early and late negative TnI value was found. In 21%, TnI increased soon after CT and normalized one month later. In the remaining 9% of patients, TnI positivity was still detectable one month later.

The TnI behaviour after CT allowed for the identification of a different cardiac risk, according to the three distinct TnI patterns. Patients without TnI elevation after CT had a good prognosis. Indeed, no significant reduction in LVEF was observed in this group, and a very low incidence of cardiac events (1%) occurred during the more than three-year long follow-up. Hence, in consideration of the high-negative predictive value of troponin (99%), low-risk patients (70% of patients in this study) that do not require close cardiac surveillance after CT may accurately be identified. In contrast, TnI positive patients had a greater incidence of major adverse cardiac events. Among TnI positive patients, the persistence of the TnI increase one month after CT is consistent with a greater cardiac impairment and a higher incidence of cardiac events than in patients showing only a transient increase (84% and 37%, respectively; p<0.001) [31].

TnI increase is also detectable in patients undergoing standard dose of chemotherapy. Experience in our institute shows increased TnI value in 20% of patients treated with schedules containing adriamycin-cyclophosphamide (AC); however, TnI increases, even less frequently (10–15%), also after the administration of schemes considered less cardiotoxic. More recently, we found TnI increase also in patients treated with monoclonal antibodies (20%, 5% and 8% in trastuzumab, bevacizumab and rituximab treated patients, respectively). In all patients, the prognostic relevance of TnI increase, in terms of LVEF reduction, was confirmed. In our view, TnI can be proposed as a golden standard marker for the assessment of cardiac safety of both old and new antineoplastic treatments, regardless of the mechanism underlying the cardiac toxic effect.

## Haemodynamic markers: NT-proBNP

Natriuretic peptides have recently emerged as biomarkers potentially useful in the diagnosis and prognostic stratification of patients with heart failure. Particularly, NT-proBNP is released from the heart in response to a cardiac overload [32].

**Figure 4:**
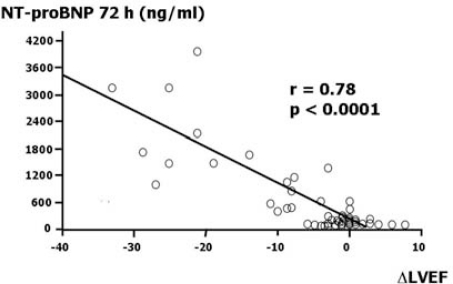
Scatterplot of N-terminal-proB-type natriuretic peptide (NT-proBNP) value at 72 hours against left ventricular ejection fraction (LVEF) changes at 12 months versus baseline

We evaluated the usefulness of NT-proBNP as an early marker able to predict cardiac dysfunction in patients affected by aggressive malignancies who were treated with high-dose CT [33]. We found three distinct NT-proBNP concentration patterns. Thirty-one per cent of patients had no changes in NT-proBNP concentrations during the six samples taken in the 72 hours after CT; 35% of patients had only a transient increase, with concentrations normalizing at 72 hours. In all these patients, no significant echocardiographic changes were recorded during follow-up. Thirty-three per cent of patients with persistently increased NT-proBNP concentrations at 72 hours developed a significant worsening of both diastolic and systolic properties values during the 12 months of observation. In particular, the echocardiographic monitoring revealed significant increases in mitral deceleration time, in isovolumetric relaxation time and in mitral E/A ratio. LVEF mean value decreased from 62.8% to 45.6% (p<0.001). We also found a strong relationship between NT-proBNP value at 72 hours, and LVEF changes at 12 months versus baseline (Figure 4).

Like TnI, NT-proBNP seems able to give us two kinds of information; the first is qualitative: it is able to identify patients who will develop cardiac dysfunction after CT; the second is quantitative: the absolute value of NT-proBNP at 72 hours reflects the degree of the future left ventricular impairment. The innovative aspect of these markers is that they give us this information at a very early phase, soon after the CT administration, and long before a functional impairment has occurred and could be identified by diagnostic techniques such as echocardiography.

Further prospective studies are needed to clarify whether both markers, TnI I and NT-proBNP, give the same kind of information, or whether their combination permits better stratification of the cardiac risk of cancer patients treated with CT.

## Cardiotoxicity prevention: a new prophylactic approach

The possibility of identifying patients at higher risk of developing cardiotoxicity by cardiac biomarkers provides a rationale for the development of targeted preventive pharmacological strategies directed at counteracting cardiac dysfunction and cardiac complications occurrence.

Considering the results of our published studies, a possible clinical application of these markers could be the evaluation of pharmacological strategies in selected high-risk patients, with the aim of preventing acute cardiac damage, left ventricular dysfunction and cardiac events. Two different therapeutic strategies could be implemented in order to reduce the clinical impact of cardiotoxicity: (1) use of specific cardiologic treatments given to cancer patients during CT in the attempt to prevent or blunt the rise of these markers; (2) use of cardiologic treatments given only to selected cancer patients, showing an increase in these markers after CT. This, with the aim of interfering with the natural evolution of cardiac toxicity and prevent the occurrence of left ventricular dysfunction and adverse cardiac events. We hypothesize that cardioprotective therapies that might limit or prevent marker rise after CT, as well as cardiologic treatments that interfere with their persistence, could improve cardiac prognosis of these patients.

Angiotensin-converting enzyme inhibitors (ACEI) have been shown to slow the progression of left ventricular dysfunction in several different clinical settings, including AC-induced CMP [34,35]. Furthermore, data referring to experimental models suggest that the cardiac renin-angiotensin system (RAS) plays an important role in the development of AC-induced CMP, and that treatment with ACEI may protect against CT-induced cardiotoxicity [36–41]. According to these data, a prophylactic strategy based on the use of ACEI could prevent cardiotoxicity in selected high-risk patients.

**Figure 5:**
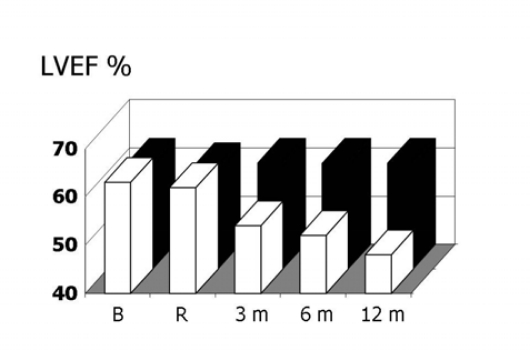
Left ventricular ejection fraction (LVEF) at baseline (before chemotherapy) and during 12-month follow-up in enalapril patients (solid bars) and controls (open bars). B = baseline; R = randomization to enalapril or no therapy (one month after chemotherapy); p value for repeated measures analysis of variance < 0.001

In a recent study from our institute, 473 consecutive cancer patients undergoing high-dose CT were evaluated; of them, 114 (24%) showed TnI increase soon after CT and were randomized, either to receive enalapril (ACEI-group, *n*=56), or not (controls, *n*=58) [42]. Treatment, started one month after CT, was continued for one year. In ACEI-group, LVEF did not change during the treatment period (Figure 5), and a lower incidence of adverse cardiac events was observed (Table 1).

**Figure 6:**
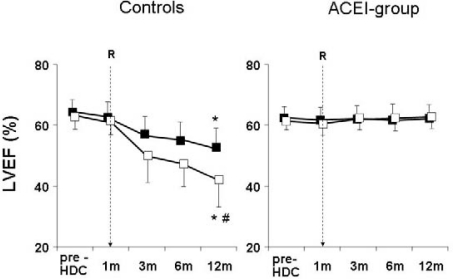
**Left ventricular ejection fraction at baseline and during the 12 month follow-up in controls (left panel) and in ACEI-group (right panel), in patients with (open squares) or without (solid squares) persistent troponin I (TnI) increase (R = randomization)** *p<0.001 versus baseline and randomization for all time points; # p<0.001 versus patients without persistent TnI increase. p value for treatment effect <0.001. p value for effect of persistent TnI increase <0.001. p value for interaction between treatment and persistent TnI increase < 0.001. (From Cardinale et al [42])

The LVEF was also analysed separately in patients with only transient TnI increase, and in those with persistent increase. In agreement with previous findings [31], untreated patients with a persistent (one month after the end of CT) TnI increase had a greater long-term LVEF reduction than patients with only transient TnI increase (LVEF decreased from 62% to 43% in the former group and from 63% to 57% in the latter; p<0.001). In treated patients, the benefit of enalapril was present in both subgroups: in no patient was LVEF significantly changed (from 61% to 62% and from 62% to 61%, respectively; p=NS), confirming that patients with persistent TnI increase are at particularly high risk of cardiotoxicity and may particularly benefit from this preventive therapy (Figure 6) [42].

Although the underlying mechanisms by which ACEI may prevent cardiotoxicity and improve outcome in high-risk CT-treated patients are not completely clear, the induction of a more favourable haemodynamic condition and RAS activation counteraction are likely to play crucial roles. Local inhibition of cardiac ACE could also be involved. Data referring to experimental models suggest that cardiac RAS plays an important role in the development of AC-induced CMP, and that beneficial effects of ACEI in AC-treated animals depends on inhibition of cardiac ACE [36–41]. Moreover, treatment with lisinopril, started after the end of CT, significantly inhibited cardiac ACE activity and improved mortality, cardiac remodelling and cardiac dysfunction in an animal model [36]. Finally, increased oxidative stress has been indicated as a possible primary mechanism in the development of AC-induced cardiac toxicity, and ACEI have been shown to exert antioxidant effects by scavenging free radicals [38].

**Figure 7:**
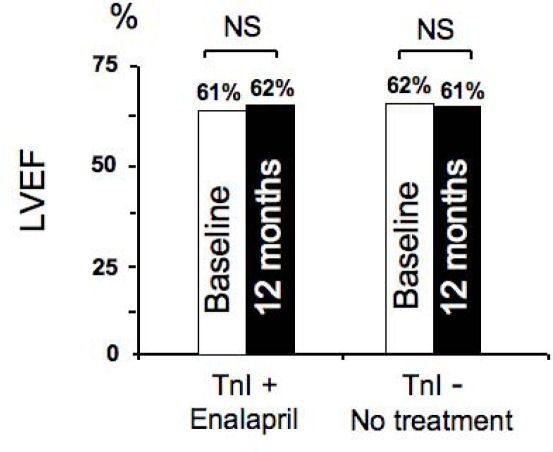
Left ventricular ejection fraction at baseline and 12 months after chemotherapy in troponin I positive patients (TnI+) treated with enalapril and troponin I negative patients (TnI −).

After this study experience, we applied the new strategy to our daily clinical practice in our institute. The results are summarized in Figure 7. We considered more than 300 patients, we found TnI positivity in 20% of patients and we started treatment with enalapril in all cases. In patients without increase of the marker, we did not perform any preventive treatment, and, as expected, no significant changes in LVEF occurred during one-year follow-up. Similarly, in patients showing an early TnI increase, we did not observe a significant reduction of systolic function. This finding confirms that cardiotoxicity can effectively be prevented in high-risk patients with this approach, and this preventive strategy can be easily applied in daily clinical practice.

## Cardiotoxicity treatment and monitoring

The most common clinical presentation of cardiotoxicity is a dose-dependent CMP leading to chronic heart failure (HF), frequently occurring after administration of CT including AC [10,11].

The prevalence of CT-induced CMP is not well known, as most studies and registries have not specifically analysed this CMP among the several possible causes of acute and chronic HF. From among the few studies in which the aetiology of HF has been evaluated in detail, a prevalence of 1% of all cases of CMP has been reported [43,44]. However, as reported previously, data from recent oncology literature indicate that more than half of all patients exposed to AC will show some degree of cardiac dysfunction ten to 20 years after CT, and 5% will develop overt HF. As more than 60,000 patients are treated every year with AC in the United States, the overall incidence of this complication is probably greatly underestimated [7].

Moreover, patients with CMP due to AC are usually considered to have an especially poor prognosis, in comparison with other more frequent forms of CMP [43], with a long-term mortality, such as ischemic and primitive CMP. The relative risk of death in patients with AC-induced CMP is reported as 2.6-fold greater than primitive CMP.

Historically, CT-induced CMP is believed to be refractory to conventional therapy, but many of these data are anecdotal and are based on reports including small numbers of patients. In addition, cancer patients with CT-induced CMP have systematically been excluded from large randomized trials evaluating the efficacy of recommended HF therapy.

As a consequence, evidence-based recommendations for management of cancer patients with asymptomatic and symptomatic CT-induced CMP are still lacking, and no definite guidelines are currently adopted. In particular, there is no evidence as to whether the use of ACEI and beta-blocker agents, recommended by international cardiologic guidelines for treatment of HF, can be directly transferred with similar long-term benefits to this particular setting.

In addition, it is very difficult to obtain evidence-based indications from the existing literature, as only an overall adult population of 108 patients can be derived from a total of 11 previous publications, mostly retrospective, without predefined end points, and in which a non-uniform treatment was considered [20,43–52].

In these studies, most patients were treated with digitalis and diuretics, 30% of patients were treated with ACEI, 5% with beta-blockers, and only 23% of patients received a combination of beta-blockers and ACEI. Therefore, even if in some cases patients experienced an improvement of systolic function and relief of symptoms, the anecdotal nature of these observations does not allow us to derive clear indications, in terms of defining the best therapeutic strategy for this form of CMP.

Furthermore, an open critical question remains: whether or not to treat cancer patients with asymptomatic left ventricular dysfunction [7,20,43–52].

Due to the different aetiology and age distribution of this kind of CMP, when compared with the more frequent ischemic or idiopathic CMPs, there is some concern as to whether the use of ACEI and beta-blocking agents can be directly applied to a cancer patient setting. One of the trickier aspects of the late cardiac dysfunction associated with AC is the early asymptomatic nature of the disease. Given the silent nature of the underlying state, many authors have suggested only screening programmes to look for overt HF, and, at present, CT-induced CMP patients are treated only if symptomatic. A crucial issue is whether or not, and eventually how, to treat asymptomatic patients in whom left ventricular dysfunction is detected on routine screening examinations. To date, there is no consensus about what (if anything) can be done to curtail the progression of CT-induced CMP [7]. It is likely that the typical medications used for HF are highly effective, but there may be special concern in cancer patients and, conventionally, the tendency is not to treat aggressively [53].

Preliminary unpublished data from our institute suggest that the time elapsed from the end of CT to the start of HF therapy, with ACEI and, when tolerated, with beta-blocking agents, is a crucial variable for recovery of cardiac dysfunction. Indeed, the likelihood of obtaining a complete LVEF recovery is higher in patients in whom the treatment is initiated within two months from the end of CT. After this time limit, however, this percentage progressively decreases and no complete LVEF recovery is observed after six months.

On the basis of these data, we can speculate that, in most previously published studies, the poor response to therapy was possibly due to the under-use of modern drugs like ACEI and beta-blocking agents, and to the long (>12 months) time elapsed from the end of CT to the beginning of cardiac treatment, that is, when cardiac damage was no longer reversible. This emphasizes the crucial importance of an early detection of cardiotoxicity and suggests that an aggressive approach based on the association of both ACEI and beta-blocking agents should always be considered, and attempted, in all cases of CT-induced CMP.

Although preventing AC-induced cardiotoxicity remains the optimal target, cardiac function should also be monitored, in patients receiving potentially cardiotoxic CT, in order to detect early cardiac abnormalities while they are still reversible. Indeed, the American College of Cardiology, the American Heart Association, and the American Society of Echocardiography recommend baseline routine evaluations and recurrent re-evaluations by echocardiography in patients undergoing CT [54]. However, this recommendation is often disregarded in asymptomatic patients and in those recovered from the oncologic disease. Oncologists and cardiologists should plan these assessments jointly, sharing therapy decisions and monitoring programmes, in order to prevent both potentially fatal oncologic and cardiologic diseases.

## Cardio-oncology: conclusion

Cardio-oncology is a novel, interdisciplinary, rapidly evolving area of growing interest, based on a comprehensive approach for the management of cancer patients with cardiac diseases. Due to the lack of evidence-based indications and to the urgent need for expertise in this setting, cardio-oncology represents a novel, topical research and clinical field, largely unexplored. Involved clinicians and researchers have the ambitious task of investigating this setting, outlining new evidence-based guidelines. This represents a big challenge for both cardiologists and oncologists, and, at the same time, is a stimulating incentive.

## Figures and Tables

**Table 1:**
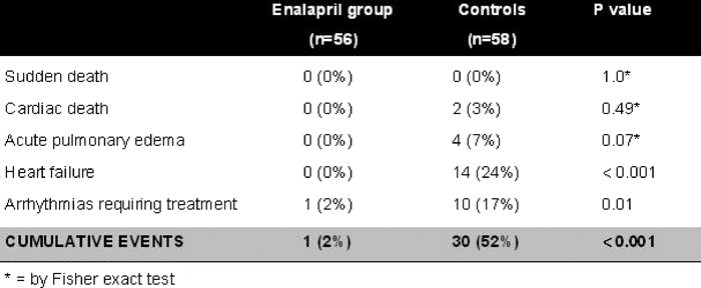
Cardiac events during the one-year follow-up in enalapril-treated patients and in controls (modified from Cardinale *et al* [42])
